# Pandemic-proof recruitment and engagement in a fully decentralized trial in atrial fibrillation patients (DeTAP)

**DOI:** 10.1038/s41746-022-00622-9

**Published:** 2022-06-28

**Authors:** Ashish Sarraju, Clark Seninger, Vijaya Parameswaran, Christina Petlura, Tamara Bazouzi, Kiranbir Josan, Upinder Grewal, Thomas Viethen, Hardi Mundl, Joachim Luithle, Leonard Basobas, Alexis Touros, Michael J. T. Senior, Koen De Lombaert, Kenneth W. Mahaffey, Mintu P. Turakhia, Rajesh Dash

**Affiliations:** 1 Division of Cardiovascular Medicine & Cardiovascular Institute, Stanford University School of Medicine, Palo Alto, California USA; 2 Center for Digital Health, Stanford University School of Medicine, Palo Alto, CA USA; 3 Stanford Center for Clinical Research (SCCR), Palo Alto, CA USA; 4 Bayer AG, Wuppertal, Germany; 5 Huma Therapeutics Ltd, London, UK; 6 Yuzu Labs PBC, San Jose, CA USA; 7 VA Palo Alto Health Care System, Palo Alto, CA USA

**Keywords:** Clinical trials, Arrhythmias

## Abstract

The Coronavirus Disease 2019 (COVID-19) pandemic curtailed clinical trial activity. Decentralized clinical trials (DCTs) can expand trial access and reduce exposure risk but their feasibility remains uncertain. We evaluated DCT feasibility for atrial fibrillation (AF) patients on oral anticoagulation (OAC). DeTAP (Decentralized Trial in Afib Patients, NCT04471623) was a 6-month, single-arm, 100% virtual study of 100 AF patients on OAC aged >55 years, recruited traditionally and through social media. Participants enrolled and participated virtually using a mobile application and remote blood pressure (BP) and six-lead electrocardiogram (ECG) sensors. Four engagement-based primary endpoints included changes in pre- versus end-of-study OAC adherence (OACA), and % completion of televisits, surveys, and ECG and BP measurements. Secondary endpoints included survey-based nuisance bleeding and patient feedback. 100 subjects (mean age 70 years, 44% women, 90% White) were recruited in 28 days (traditional: 6 pts; social media: 94 pts in 12 days with >300 waitlisted). Study engagement was high: 91% televisits, 85% surveys, and 99% ECG and 99% BP measurement completion. OACA was unchanged at 6 months (baseline: 97 ± 9%, 6 months: 96 ± 15%, *p* = 0.39). In patients with low baseline OACA (<90%), there was significant 6-month improvement (85 ± 16% to 96 ± 6%, *p* < 0.01). 86% of respondents (69/80) expressed willingness to continue in a longer trial. The DeTAP study demonstrated rapid recruitment, high engagement, and physiologic reporting via the integration of digital technologies and dedicated study coordination. These findings may inform DCT designs for future cardiovascular trials.

## Introduction

Randomized clinical trials remain the bedrock of scientific evidence to evaluate new therapeutic approaches. Large-scale phase 3 clinical trials typically rely on manually driven recruitment and data collection visits conducted through specific coordinating centers. Such trials require burdensome financial, operational, and time commitments from investigators and participants; yet, they frequently fail to meet their enrollment targets on time, leading to costly delays and compromising statistical power. Up to 86% of clinical trials do not achieve their enrollment targets in a timely fashion^1,2^. The Coronavirus Disease 19 (COVID-19) pandemic has further constrained traditional trial recruitment and execution procedures worldwide in an unprecedented manner, leading to widespread disruption of clinical trial efforts^3^. Patient enrollment in clinical trials per site in April 2020 was reported to be 80% lower compared with April 2019. There remains a significant need for novel clinical trial designs to achieve adequate enrollment and successful execution in a timely manner^4,5^.

Decentralized trials broadly refer to trials wherein recruitment and data collection procedures are not restricted to fixed locations. Trial activities can be done by participants where they are, including at home, using remote technologies, thus potentially increasing interest and improving the participant journey in the study^4^. Compared with traditional trial designs, decentralized protocols may offer an opportunity to scale recruitment and transform trial conduct while maintaining engagement and high-quality results. There is a strong need to develop validated, feasible, decentralized clinical trial frameworks to facilitate remote study conduct while also reducing the costs and time associated with trial execution. Rapidly emerging digital health technologies may help remotely obtain the objective physiologic data that cardiovascular (CV) trials often require. However, no large-scale phase 3 CV trial has employed a 100% remote, decentralized protocol including physiologic measurements. Whether decentralized protocols can be successfully used for large, pivotal CV drug trials remains uncertain, and it remains vital to validate the feasibility of adopting decentralized protocols for CV trials before large-scale implementation.

We, therefore, sought to validate the feasibility of a completely virtual, decentralized clinical trial for CV interventions by integrating a range of individual digital health technologies for physiologic measurements among atrial fibrillation patients on oral anticoagulation (OAC) therapy. We hypothesized that the decentralized protocol would achieve rapid recruitment, robust protocol adherence, and high participant engagement.

## Results

### Study cohort

**Fig. 1:**
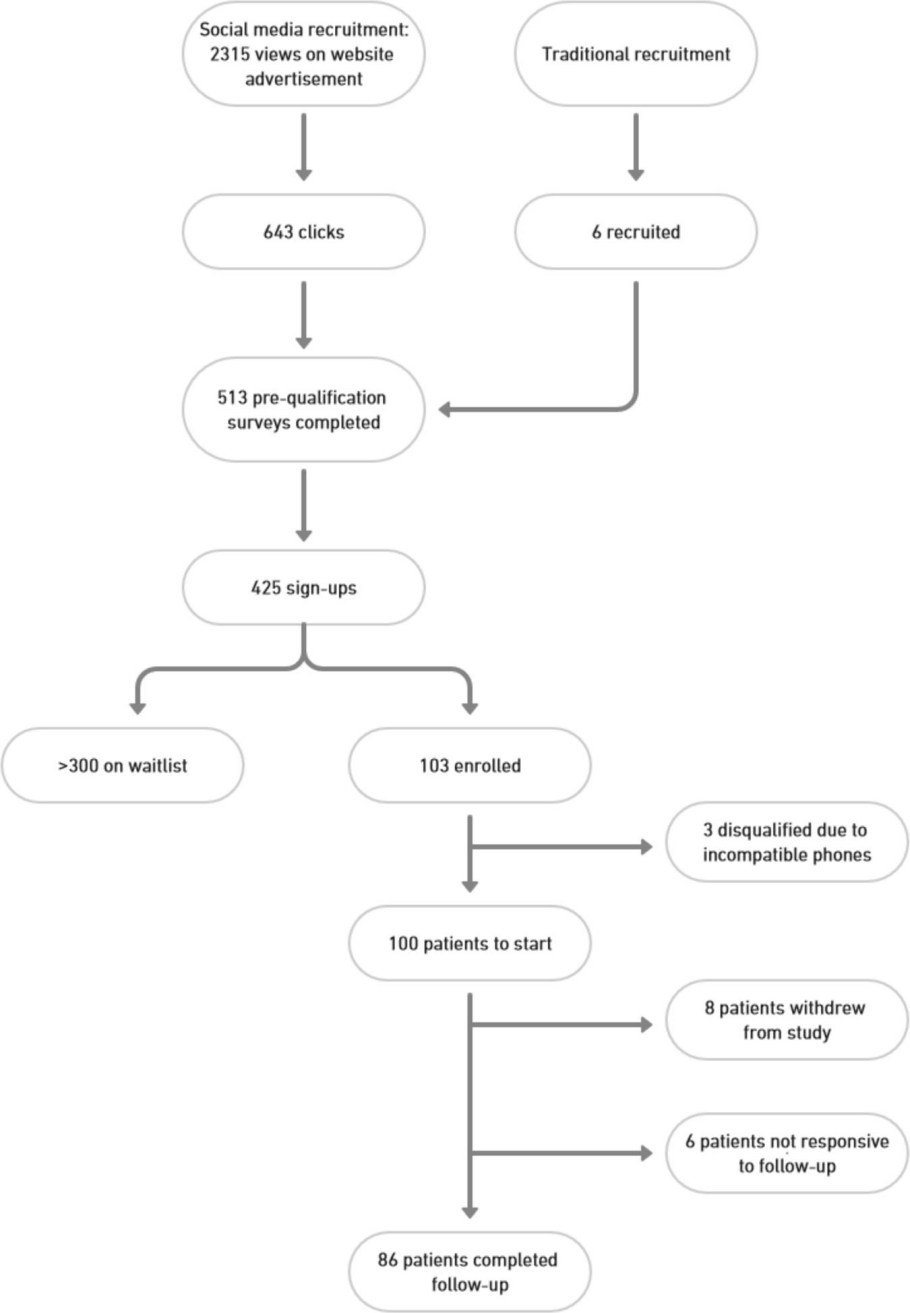
DeTAP recruitment and enrollment flow. Participants were recruited through both traditional and social media methods. This figure outlines the flow of participant recruitment and enrollment.

A total of 100 patients were enrolled, with a mean age of 70 ± 15 years and with 44% women, 90% White, 3% Black, and 4% Asian participants (Fig. 1 and Table 1). This was the first clinical trial for 78% of participants, and 56% reported never owning a Bluetooth-connected health device before this study. Other characteristics are described in Table 2.

**Table 1:** Baseline patient characteristics.

Characteristic	Baseline (*N* = 100)	6 months (*N* = 86)
Age (years)	70+/−15	68+/−12
Female (%)	44	44
Race/Ethnicity		
White (%)	90	85
Black (%)	3	3.5
Asian (%)	3	3.5
Stanford Healthcare affiliation (past or present, *N*)	12 (6 through virtual recruitment)	11
Urban dwelling (%)	81	73
Education level at bachelor’s degree or more (%)	73	66
Annual income $50,000 or more (%)	64	54
Oral anticoagulant use (*N*, %)	97 (97%)	86 (100%)
Vitamin K antagonist/warfarin (*N*, %)	14 (14%)	1 (1%)
Rivaroxaban (*N*, %)	26 (26%)	21 (24%)
Apixaban (*N*, %)	39 (39%)	35 (41%)
Dabigatran (*N*, %)	17 (17%)	17 (20%)
Other (*N*, %)	1 (1%)	1 (1%)

**Table 2:** Overview of study visits, technologies used, and remote data collection procedures.

Study visit	Technology	Administered by	Data collected
Baseline	Study app	Patient	Surveys: Medical History, OAC adherence, PAM-13, telehealth
	Remote devices	Patient	Blood pressure, Electrocardiogram
	Live televisit	Investigator	Protocol review, remote device verification
Weekly	Study app	Patient	Five-question survey: OAC adherence, adverse symptoms
Every 6 weeks (+/− 1 week)	Remote devices	Patient	Blood pressure, Electrocardiogram
	Live televisit	Investigator	Review protocol and instructions, address questions
Study midpoint (End of month 3)	Study App	Patient	Surveys: OAC adherence, Nuisance bleeding
	Live televisit	Investigator	Review protocol and instructions, address questions
Study End (End of month 6)	Study App	Patient	Surveys: Medical History update, OAC adherence, Nuisance bleeding, PAM-13, telehealth
	Live televisit	Investigator	Obtain patient engagement, satisfaction, feedback, and interest in future participation

### Recruitment metrics

**Fig. 2:**
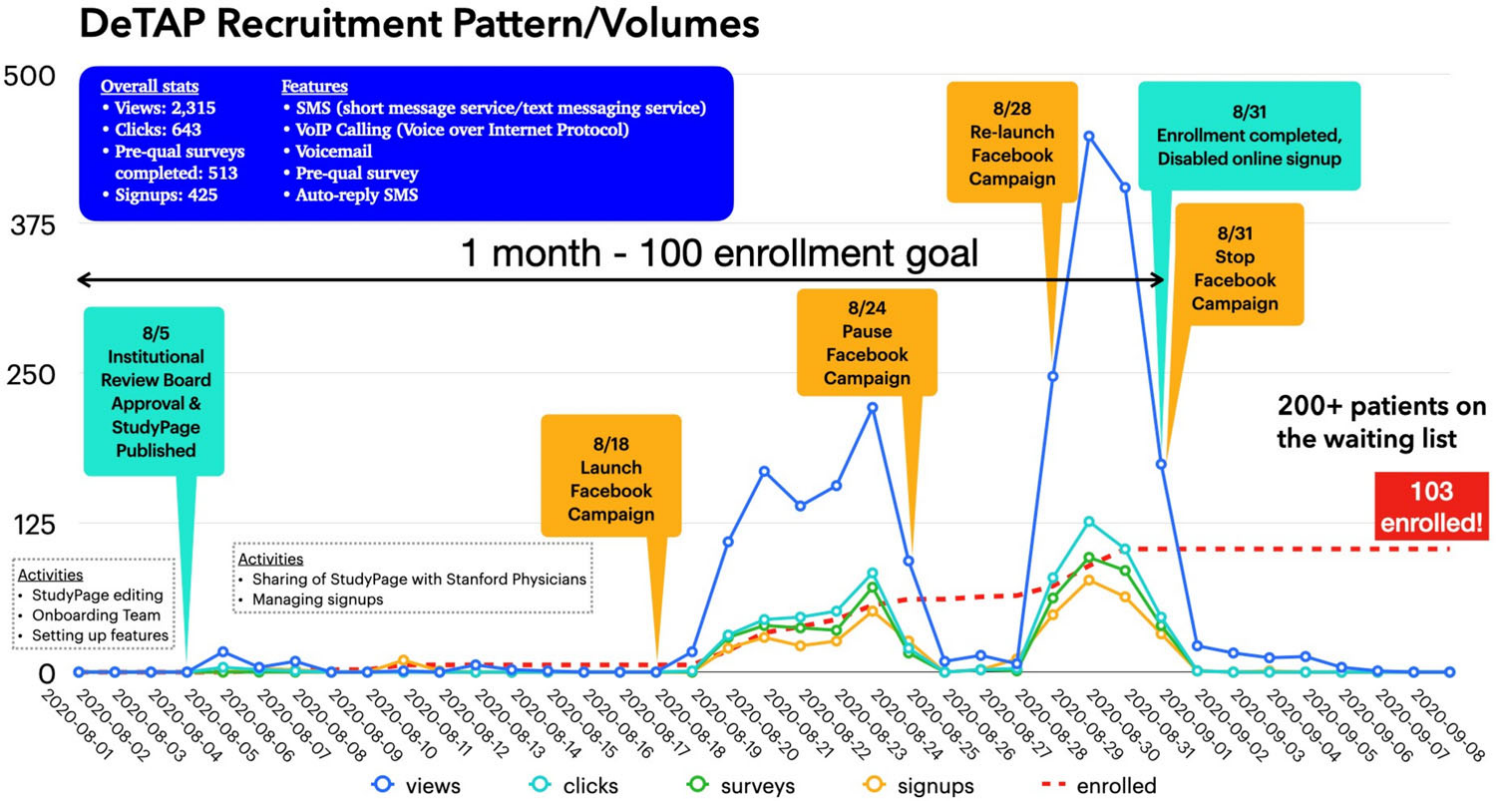
DeTAP trial recruitment volumes over time with traditional and virtual, social media-based recruitment. IRB Institutional Review Board, FB facebook, SMS short message service (text messaging service), VoIP Voice over internet protocol (Figure provided by StudyPages, Yuzu Labs PBC).

In-person recruitment was first initiated on August 6, 2020, achieving total recruitment of six patients during the entire study. On August 19, social media-based recruitment began, which led to a dramatic recruitment surge, completing recruitment, consent, and enrollment of 94 patients over 12 days and more than 300 eligible patients on the study waitlist (Fig. 2).

### Protocol engagement

Of 100 enrolled patients, 86 completed 6 months of follow-up. A total of eight patients withdrew from the study, while six patients were not responsive to follow-up.

The primary endpoint of televisit completion rate was 91%, corresponding to greater than 335 total visits. Of 86 patients who completed 6 months of follow-up, 85 patients (98.8%) acquired four or more electrocardiograms (ECGs) and blood pressure (BP) measurements in accordance with the study protocol. Oral anticoagulation (OAC) survey completion proportion was 85% for the study overall, ranging from 55% to 96% completion during any given week.

Patient activation measure scores, a secondary outcome of engagement, remained high during the study (baseline: 70%, 6 months: 74%, *p* = 0.32).

### OAC adherence

The primary endpoint of OAC adherence was unchanged overall during the study (baseline: 97 ± 9%, 6 months: 96 ± 15%, *p* = 0.39, Table 3). Among patients with low baseline OAC adherence, there was significant improvement during the study at 6 months (baseline: 85 ± 16%, 6 months: 96 ± 6%, *p* = 0.002).

**Table 3:** Key survey outcomes at baseline and study end.

Survey	Baseline	6 months
Primary outcomes		
OAC adherence, overall (%)	97 ± 9	96 ± 15
Secondary outcomes		
OAC adherence, low baseline (%)	85 ± 16*	96 ± 6*
Nuisance bleeding positive (%)	73 (3 months)	57
Patient Activation Measure (PAM-13) score (%)	70	74

*p = 0.002.

### Bleeding

A total of 62 participants (72%) reported nuisance bleeding at 3 months and a total of 47 participants (57%) reported nuisance bleeding at the end of the study.

### Patient feedback

At the end of the study, when asked about their willingness to participate in a longer DCT of 12–18 months, 69 out of 80 respondents (86%) expressed some willingness (69 answered agree or strongly agree, and 6 were neutral), while 5 (6%) disagreed or strongly disagreed. Qualitative, free-text patient feedback on the study indicated favorable feedback regarding atrial fibrillation education, the ability to use devices at home for a study, regular contact with study coordinators, and improved medication adherence. Patients reported study app usability, hardware/device pairing issues, lack of available ECG interpretations, and lack of understanding of the long-term purpose of this study as potential issues.

## Discussion

The DeTAP trial demonstrates the feasibility of a potentially scalable, fully decentralized CV intervention trial requiring remote physiologic measurements among participants with atrial fibrillation, with robust social media-based recruitment and strong engagement. Participants successfully self-administered and transmitted physiologic BP and ECG measurements through remote devices and completed regular surveys and televisits remotely. Overall OAC adherence was high at baseline and did not significantly change during the study. 86% of participants completing the end of study survey expressed willingness to continue for a longer study duration via our protocol.

Timely enrollment remains a recurring challenge for large-scale phase 3 CV trials, often requiring multiple, adaptive, and resource-intensive recruitment strategies to achieve adequate statistical power^5^. Our findings point to the potentially beneficial role of virtual recruitment, as we observed a dramatic surge in enrollment upon transitioning from traditional in-person recruitment to a social media-based virtual strategy. At completion, the study had a waitlist population of eligible participants that was more than twice our target enrollment. The speed and geographic reach of virtual recruitment—along with the ability to refine advertisement formatting and targeting to achieve maximal views—may have helped achieve timely recruitment.

These findings align with trends observed in contemporary, foundational studies that employed direct-to-participant designs including the mHealth Screening to Prevent Strokes (mSToPS) trial, the Apple Heart Study, the Fitbit Heart Study, and the Mobile Health [mHealth] Technology for Improved Screening, Patient Involvement and Optimizing Integrated Care in Atrial Fibrillation (MAFA II) study^6–9^. These key studies leveraged remote procedures and achieved large-scale (thousands of patients) recruitment and conduct, including a self-applied continuous ECG monitoring patch in the mSToPS trial, and wearable devices in the Apple Heart Study, the Fitbit Heart Study, and the MAFA II study.

We designed our protocols to optimize the speed of recruitment, but such approaches could potentially be adapted to focus on crucial alternative trial metrics, including participant diversity. Similar protocols could be adapted across regions in the United States and other countries where patients use the same social media tools that were employed in this study. Major CV clinical trials, including guideline-informing trials of atrial fibrillation, often underrepresent certain racial/ethnic groups, indicating systemic bias in traditional trial recruitment^10^. Potential barriers to diverse trial recruitment include mistrust, low comfort with the research process, lack of adequate information for decision-making, and a lack of understanding of the value of the research^11^. The time and resource constraints of traditional trials have also been cited as key barriers, which may be overcome by decentralized conduct. Accommodating the language needs of diverse populations may be crucial as well^12^.

Prespecifying the desired representation of diverse groups and creating tailored recruitment plans for stratified enrollment to address such barriers may help to improve diverse participation. For example, the Systolic Blood Pressure Intervention Trial (SPRINT) of intensive systolic blood pressure lowering prespecified an enrollment goal of 40% racial/ethnic minority patients^5^. The SPRINT recruitment team exceeded their goal by utilizing targeted mailing campaigns, culturally sensitive messaging, language translation, as well as geographic targeting in locations where minority representation was high. Such successful strategies should be adapted to social media approaches that could offer wider geographic capture, potentially lower cost, and higher flexibility in refining recruitment materials for maximum engagement to achieve diverse recruitment. Researchers should explore search engine optimization based on a stratified enrollment schema. In doing so, researchers should ensure that recruitment does not reflect or exacerbate the digital divide, that is, the differential access to technologies in low-resource communities, older patients, and diverse racial/ethnic populations^13–15^. As seen in the present study, the lack of prespecified stratified enrollment and targeted recruitment efforts can lead to less diverse representation due to the above-mentioned barriers and the digital divide. Patient representatives and/or community participatory research experts should be included in trial leadership and advisory roles to help engage communities in the recruitment and design process. The potential of virtual approaches to achieve representative patient populations should be studied in dedicated efforts.

We used two FDA-approved devices for BP and ECG measurements and provided remote, primarily asynchronous training to participants. High completion rates of remote BP/ECG measurements indicated that physiologic measurements were successfully obtained through these approaches with low attrition. We did not identify critical issues that could prevent the scalability of these approaches to larger trial populations.

While our remote technologies focused on vital sign and rhythm analysis, there is growing contemporary interest in validating novel “digital biomarkers” using biosensor-derived data for complex conditions such as diabetes, heart failure, knee injury, and Parkinson’s disease^16–19^. As such evidence accumulates, validated remote monitoring systems may become increasingly relevant for decentralized trial conduct.

Overall, OAC adherence was high among study participants and did not change significantly during the study. Among those with lower self-reported OAC adherence rates at baseline, OAC adherence improved during the study. Complex interventions that include patient reminders and communication through phone calls have been previously shown to improve short-term medication adherence^20^. Several study interventions requiring low-resource utilization, including regular surveys, smartphone notifications, and televisits, potentially increased OAC adherence.

While our study was limited to 6 months of follow-up, at the end of our study, >80% of participants expressed willingness to continue for a 12 to 18-month protocol. Our experience may thus inform fully decentralized designs for longer, pivotal CV intervention trials that may need remote physiologic measurements and outcome tracking while maintaining siteless recruitment and high engagement.

Our study combined remote technologies and virtual deployment with dedicated remote human coordinator “checkpoints”, providing patient education and reinforcing trial protocols. As digital technologies rapidly evolve, dedicated study coordination may remain an important part of decentralized pivotal CV trial designs to provide the necessary level of technical support and education across diverse trial participants to ensure the collection of research-grade data with low missingness. While remote coordination will likely be less cumbersome than in-person coordination, the scalability and utility of dedicated human coordination in larger trial populations should be assessed in follow-up studies.

Our study had certain limitations. The majority of our participants were White and urban dwelling, and nearly 70% of our population had bachelor’s degrees or higher levels of education, which limits the generalizability of our findings. Future efforts should develop and validate decentralized protocols in more diverse populations to ensure that the use of digital technologies does not exacerbate existing health inequities. Adapting recruitment strategies to focus on participant diversity as a key trial metric may help address this goal. OAC adherence was fully self-reported and was not validated objectively by study investigators.

In conclusion, we report the feasibility of a fully decentralized, potentially scalable CV intervention trial including remote physiological monitoring among patients with atrial fibrillation, with robust virtual recruitment, strong engagement, and successful tracking of OAC adherence. These findings may provide a foundation to inform the design and conduct of future decentralized protocols for CV trials.

## Methods

### Study design

**Fig. 3:**
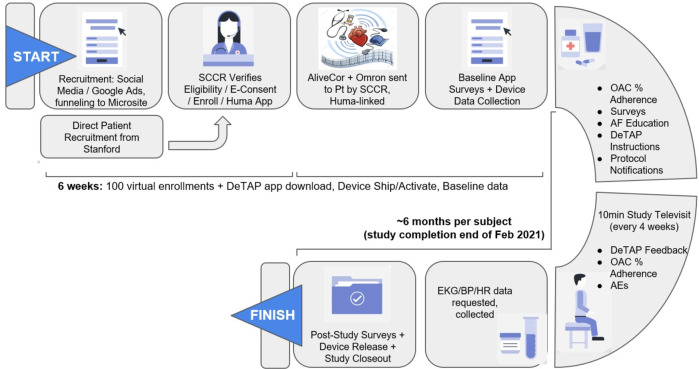
DeTAP study protocol flow. SCCR Stanford Center for Clinical Research, OAC oral anticoagulant, EKG electrocardiogram, BP blood pressure, HR heart rate, DeTAP decentralized trial in afib patients, App smartphone/mobile application, AF atrial fibrillation, AEs adverse events.

This was a single-arm, 6-month-study (Clinicaltrials.gov identifier NCT04471623) to validate the feasibility of an integrated suite of digital health technologies to achieve rapid recruitment and protocol adherence through a fully decentralized trial protocol. The study was sponsored by Bayer Pharmaceuticals and coordinated and conducted by an academic research organization, the Stanford Center for Clinical Research (SCCR). Recruitment was initially planned to be conducted primarily through social media-based strategies for the whole duration of the study, while supplemented by traditional in-person methods, to achieve an enrollment goal of 100 atrial fibrillation patients over age 55 years on OAC therapy. However, based on Stanford University Institutional Review Board (IRB) recommendations, participants were recruited and enrolled first solely through traditional on-site methods. This process consisted of local communication with local cardiology providers and a review of clinic rosters for eligible patients, followed by direct phone outreach to eligible patients identified for screening and enrollment. Following 2 weeks of traditional study recruitment with the recruitment of six total patients, the IRB allowed the initiation of the social media-based strategy for the remainder of the study to augment recruitment. Social media recruitment strategies were developed that targeted adults living in California, 55 years of age or older, with an interest in cardiovascular health topics related to atrial fibrillation and heart disease, and using an iPhone 6S and above or an Android 6.0 and above. Advertisements (ads), including text, headlines, and images, were created to maximize engagement and micro-website signups. Over time, the ad algorithm learned which ads performed best and delivered those ads more frequently than the others. Regardless of whether the subject was initially contacted via traditional or social media outreach, all interested participants went through a common, 100% decentralized pathway for eligibility qualification, consent, enrollment, and study execution (Fig. 3).

Whether subjects were contacted via traditional recruitment outreach or via a social media ad, subjects were directed to the DeTAP study micro-website (facilitated by StudyPages by Yuzu Labs PBC, San Jose, CA, a HIPAA-compliant clinical research recruitment and engagement platform for online research conduct). The website included basic information about the study as well as five qualifying “yes/no” questions for the study: “are you >55 years old?”; “do you have a current or past atrial fibrillation diagnosis?”; “are you on anticoagulation medication?”; “do you own a smartphone?”; and “are you a Stanford HealthCare patient?”. Being a Stanford HealthCare patient was not a requirement for eligibility. Still, this information was collected to determine whether social media recruitment or traditional recruitment attracted more Stanford HealthCare patients to DeTAP. Potential subjects consented to the use of this qualifying information for evaluation before submitting their answers. If a subject answered “yes” to the first four eligibility questions, they received an automated email and text link that allowed them to upload a photo of their OAC prescription bottle. Submitted OAC prescription photos were evaluated by the study coordinator at SCCR to confirm OAC usage, after which eligible subjects were sent an SMS text message with a link to an electronic informed consent form for DeTAP.

Once consented, subjects were sent an email and text messaging link to download the DeTAP study app (iOS and Android operating systems, made by Huma Therapeutics Ltd, London UK), which served as the primary conduit for all study data entry, including surveys, BP/ECG recordings, and messaging through the mobile app. Televisits were conducted by telephone. Enrolled participants underwent regular, fully virtual study visits and data collection procedures (Fig. 3 and Table 2). We created a “waitlist” of participants at the time of recruitment but did not change participant numbers with replacements from the waitlist once the study started to assess retention, withdrawal, and loss-to-follow-up accurately. Videos and instruction documents regarding device activation and pairing were available to each patient through the study app. Live calls were scheduled if needed to complete the pairing process. Data collection included self-administered surveys and remote physiologic monitoring through connected devices including six-lead ECG data through the AliveCor KardiaMobile 6 L device and BP data through the Omron HEM-9210T/9200 T Bluetooth blood pressure monitor, all transmitted via the DeTAP mobile application. No in-person visits or measurements were performed. No medications were provided through the study protocol. The study was approved by the Stanford University IRB.

### Participants

A total of 100 patients were enrolled in this single-arm study. For inclusion, participants had to be aged 55 years or more with a patient-reported diagnosis of atrial fibrillation within the preceding year. Participants had to be actively prescribed OAC therapy (vitamin K antagonist or direct oral anticoagulation) for stroke prevention, had to be willing to use mobile applications and home devices through a smartphone for the study, and had to be agreeable to use televisits to conduct study visits. Participants were excluded if they had angina (stable or unstable) requiring cardiovascular functional risk stratification, catheterization, or intervention; non-compensated or hypervolemic congestive heart failure as determined by the treating clinician; had a condition that the study investigator believed would interfere with their ability to provide informed consent, comply with the study protocol, or place the person at undue risk from the study; or did not speak or read English.

### Intervention

The study intervention was the remote monitoring of the participant’s clinical status, OAC administration and OAC adherence through combined decentralized technologies that included televisits, self-administered surveys, ECG device, and BP monitor with remote data transmitted via the study mobile application (Huma Therapeutics Ltd). Participants were required to obtain their routine medications, including OAC therapy, through existing prescriptions, and the study protocol did not provide refills or new medications.

### Data collection and follow-up

The study follow-up period was 6 months. Data collection was achieved through integrating a range of digital technologies: a study mobile application for survey data; patient-administered BP and ECG measurements through previously described remote devices transmitted through the study mobile application; and televisits administered by study investigators (Table 2 and Fig. 3). Study surveys assessed OAC adherence and adverse symptoms weekly. Study surveys also assessed the patient’s medical history, OAC adherence, nuisance bleeding (Supplementary Table 1), adverse symptoms, and overall patient health engagement through the Patient Activation Survey (PAM-13) at baseline, study midpoint (3 months), and study end (6 months) follow-up visits. A total of four televisits were conducted at 6-week intervals. Remote blood pressure, heart rate (HR), and ECG measurements were performed by patients and transmitted through the study mobile application at 6-week intervals corresponding with the televisits. Televisits systematically collected the following data: completion of BP and ECG measurements, changes to OAC dosing regimen, OAC pills remaining and date of the last refill, and side effects. The televisits included midpoint and end-of-study visits which also reviewed the study protocol, addressed patient questions, and obtained patient feedback on the protocol, including interest in participating in future decentralized studies.

### Outcomes

The prespecified primary outcomes for this single-arm feasibility study were % completion of televisits, % completion of ECG and BP recording uploads, % completion of surveys, and change in % adherence to OAC by self-report at baseline and study end. In the initial study design, the primary outcome was intended to be a composite of these four endpoints. After study initiation but before study data review or analysis, statistician review subsequently recommended that these endpoints be assessed individually as separate endpoints rather than as a composite endpoint due to analytic challenges and decreased interpretability related to combining these outcomes into a single composite endpoint. Secondary outcomes included: change in % adherence to OAC from baseline to 6 months in those with low baseline adherence, PAM-13 survey scores at baseline and 6 months (study end), nuisance bleeding survey scores at 3 months and 6 months, telehealth survey results at baseline and at 6 months, and qualitative survey results at 6 months regarding patient feedback and interest in participating in future decentralized clinical trials. Low OAC adherence was defined as <90% in accordance with adherence thresholds in the literature based on associations with increased risk of stroke^21^.

### Sample size

A sample size of 100 was targeted. Based on prior survey data, our population of interest exhibited a 50% OAC adherence rate, with a standard deviation of 15% in baseline patient OAC engagement metrics, a primary outcome variable. As a result, an evaluable sample of 80 subjects provided 80% power to detect a difference of 10% based on a paired t-test and a two-sided alpha of 0.05. A larger enrolled sample of 100 allowed for subject attrition of up to 20%.

### Statistical analysis

Baseline characteristics were described using means and standard deviations or medians and interquartile range limits as appropriate based on the distribution for continuous variables. Numbers of subjects and percentages were used to describe categorical variables. Responses to the intervention were assessed using paired *t*-tests for continuous variables. For categorical variables, McNemar’s test was employed to assess differences in frequencies between baseline and end-of-study. All tests of statistical significance were completed using alpha = 0.05, two-sided. Statistical analyses were performed by the coordinating study team independent of the study sponsor.

### Reporting Summary

Further information on research design is available in the Nature Research Reporting Summary linked to this article.

## Supplementary information

Supplementary File

Reporting Summary Checklist

## Data Availability

The datasets analyzed during the current study are not publicly available. Due to reasonable privacy and security concerns, the underlying data are not easily redistributable to researchers other than those engaged in the current project’s Institutional Review Board-approved research collaborations. The corresponding author may be contacted for an IRB-approved collaboration.

## Code availability

There is no custom code or mathematical algorithm central to this study’s conclusion.
