# Supplementary material for: Pandemic-proof recruitment and engagement in a fully decentralized trial in atrial fibrillation patients (DeTAP)
Source: NPJ Digit Med. 2022 Jun 28;5:80. doi: 10.1038/s41746-022-00622-9 (PMC9240050; doi:10.1038/s41746-022-00622-9)
Supplement: Supplementary file 1 — Supplementary File [file 41746_2022_622_MOESM1_ESM.docx]

| Over the past 7 days, have you experienced easy bruising? |
| --- |
| On how many of the past 7 days did you experience easy bruising? |
| Please rate the severity of your easy bruising over the past 7 days. |
| Over the past 7 days, did you bleed from small cuts? |
| On how many of the past 7 days did you bleed from small cuts? |
| Please rate the severity of your bleeding from small cuts over the past 7 days. |
| Over the past 7 days, did you have small red dots, "blotches" or circles on your skin? |
| On how many of the past 7 days did you have small red dots, "blotches" or circles on your skin? |
| Please rate the severity of the small red dots, "blotches" or circles on our skin over the past 7 days. |
| Over the past 7 days, did you have bruises on your hands and arms? |
| Please rate the severity of the bruises on your hands and arms over the past 7 days. |
| Over the past 7 days, did you have bruises on your feet and legs? |
| On how many of the past 7 days did you have bruises on your feet and legs? |
| Please rate the severity of the bruises on your feet and legs over the past 7 days. |
| Over the past 7 days, did you have bruises on other parts of your body? |
| On how many of the past 7 days did you have bruises on other parts of your body? |

**Supplementary File**

**Supplementary Table 1. Nuisance bleeding survey questions.**
